# Scientific Validation of Ethnomedicinal Use of* Ipomoea batatas* L. Lam. as Aphrodisiac and Gonadoprotective Agent against Bisphenol A Induced Testicular Toxicity in Male Sprague Dawley Rats

**DOI:** 10.1155/2019/8939854

**Published:** 2019-04-14

**Authors:** Muhammad Majid, Fatima Ijaz, Muhammad Waleed Baig, Bakht Nasir, Muhammad Rashid Khan, Ihsan-ul Haq

**Affiliations:** ^1^Department of Pharmacy, Faculty of Biological Sciences, Quaid-i-Azam University, 45320 Islamabad, Pakistan; ^2^Department of Biochemistry, Faculty of Biological Sciences, Quaid-i-Azam University, 45320 Islamabad, Pakistan

## Abstract

Sweet potato (*Ipomoea batatas* L. Lam.), known as “Shakarqandi” in Pakistan, is an imperative root vegetable with large size, traditionally used as aphrodisiac, antiprostatic, anti-inflammatory, antidiabetic, cardiotonic, and anticancer agent. Present study was conducted to gauge aphrodisiac potential of* Ipomoea batatas *ethyl acetate (IPT-EA, IPA-EA) and methanol (IPT-M, IPA-M) extracts from tuber and aerial part, respectively, via behavioral and biochemical tests and their possible protective role in BPA-induced gonadotoxicity at the dose 300 mg/kg in male Sprague Dawley rats. Phytochemical analysis was done qualitatively and quantitatively through total phenolic and flavonoid content (TPC and TFC) and high performance liquid chromatographic (HPLC-DAD) fingerprinting while antioxidant profiling used multimode* in vitro *assays. To calculate sexual excitement mount latency, intromission latency, mount frequency, intromission frequency, ejaculatory latency, and postejaculatory interval were examined while for biochemical ratification semen characteristics, levels of testosterone, follicle stimulating hormone (FSH), luteinizing hormone (LH), and estradiol were measured. Gonadoprotective ability was assessed through comet assay and histomorphological examination of testes. Qualitative analysis ensured the presence of phenols, flavonoids, tannins, anthocyanin, saponins, coumarins, terpenoids, and betacyanin. Quantitatively maximal TPC (304.32±7.20 *μ*g GAE/mg dry extract) and TFC (214.77±4.09 *μ*g QE/mg DE) were estimated in IPA-EA extract. IPT-EA yielded maximum rutin (7.3±0.12) and myricetin (2.7±0.14 *μ*g/mg DE) while IPA-EA and IPA-M yielded maximum caffeic acid (4.05±0.22 and 1.92±0.17 *μ*g/mg DE, respectively) in HPLC-DAD analysis. Extracts enhanced sexual excitement, improved semen quality, levels of testosterone, FSH, LH, and estradiol, and successfully attenuated toxic effects of BPA. Levels of endogenous antioxidant enzymes (CAT, SOD, POD, and GSH) were restored and NO abundance was minimized. Significant stimulation in sexual behavior, amelioration of toxicity symptoms, elevated spermatic production, raised viability, vitalized levels of gonadal hormones, maintained endogenous enzymes, genoprotection, and reformed testicular histology endorsed* I. batatas* as a better aphrodisiac alternative and gonadoprotective agent.

## 1. Introduction

Among primary organic needs and biological drives, sexual urge stands very next to hunger, thirst, and sleep [1]. Libido, erection, orgasm, ejaculation, and detumescence are the principal events in defined order that describe normal sexual function. Any interference in the defined ordered sequence of the events in this response cycle leads to male sexual dysfunction (MSD) and erectile dysfunction (ED) [2]. ED is the inability to achieve and uphold erections for the satisfactory sexual performance. Data shows that almost 52% of men aging 40-70 are experiencing ED of varying degrees and defective sperm functions like low sperm density and motility [3]. Numeral studies have been conducted since last decades to figure out the exact reason resulting decline in sperm density and count [4]. Positive oxidative stress (OS) stands major among many of the factors causing male infertility. Physiological concentrations of reactive oxygen species (ROS) are needed by spermatozoa for their fertilization, acrosome reaction, hyperactivation, motility, and capacitation. Excessive production of ROS may lead to idiopathic male infertility, sexual dysfunction, and oxidative stress-induced sperm DNA damage [5, 6]. Plasma membrane of sperm cell comprised polyunsaturated fatty acids (PUFA) which are easily oxidized by ROS and have no robust preventive capacity and repair mechanism against them, ultimately leading to sperm death [7].

Exposure to xenobiotics and several environmental contaminants including alcohol, smoking, thioacetamide, and bisphenol A (BPA) are held responsible for the propagation of ROS level which cause disorders in all vital organs including testes [8]. BPA (2, 2-bis (4-hydroxylphenyl) propane) is one of the highly consumed chemical widely used in manufacturing of polycarbonate plastics, dental sealants and amalgams, packing resin liner of canned foods, and thermal materials [9]. Continuous exposure of BPA may lead to oxidative stress which causes genitourinary abnormalities, sperm deformity, epigenetic variations, enlarged prostate mass, and reduced epididymal weight [10]. Even in low doses (<50 mg/kg) BPA lowers sperm counts, increase sperm DNA damage, reduce testosterone levels, and lessens motility of the sperms [11, 12]. On account of inefficacy of modern medicines against oxidative stress related genitourinary disorders, the importance of searching safe and effective materials with antioxidant potential are emphasized.

Antioxidants, i.e., dietary, endogenous, and metal binding proteins protect spermatozoa from ROS producing abnormal spermatozoa, scavenge ROS, prevent DNA fragmentation, improve semen quality, reduce cryodamage to spermatozoa, block premature sperm maturation, and stimulate spermatozoa [7, 13]. Though sexual dysfunction can be treated by both medical and surgical treatment modalities but plant-derived antioxidants and aphrodisiac remedies continue to be a popular alternative for men and women [14].

Efficient food products, which are rich in antioxidants, aid specific physiological functions in addition to being nutritive. One such functional food, sweet potato (*Ipomoea batatas* L. Lam.) from family Convolvulaceae, is the 7th most important crop in the world (FAO 1997) [15]. It is an imperative root vegetable with large size, high starch contents, and sweet taste [16].* I. batatas *is rich in vitamins like pantothenic acid (vitamin B5), pyridoxine (vitamin B6), thiamin (vitamin B1), niacin and riboflavin [17], polyphenols (anthocyanin) and phenolic (caffeic, monocafeoylquinic, dicaffeoylquinic, and tricaffeoylquinic acids) [15, 18], triterpenes (*β*-carotene and boehmeryl acetate) trace elements (iron, calcium and zinc), and proteins [16]. Leaves and areal part of* I. batatas *contain more polyphenols than any other commercial vegetables such as spinach, cabbage, and lettuce. Leaves of sweet potato contain at least 15 anthocyanins and 6 polyphenolic compounds [19]. Leaves and tubers of* I. batatas* are powerful antioxidants and traditionally used as aphrodisiac, anti-inflammatory, antiprostatic, energizer, laxative, bactericidal, antifungal [20], antianemic, antihypertensive, and antidiabetic [21]. In Pakistan, it is known as “Shakarqandi” and is used against infertility, allergies, arthritis, cardiovascular problems, cancer, HIV, and ageing by folklores [22–24].

Due to functional food likeness, rich phytochemical profile, potent antioxidant capacity, nontoxic attitude of* I. batatas*, and its ethnomedicinal use as aphrodisiac and antiprostatic agent, present study has been designed to assess tubers and aerial part as a natural remedy against male sexual dysfunction and bisphenol A induced gonadotoxicity in Sprague Dawley rats.

## 2. Material and Methods 

### 2.1. Preparation of Extract

Dr. Rizwana Aleem Qureshi, Professor at Department of Plant Sciences, identified and authenticated the plants samples collected from Sargodha District. After awarding accession number (PHM-501), plant specimen was preserved at Herbarium of Pakistan, Quaid-i-Azam University, Islamabad. Plant's tubers and veins were gently washed with running tap water to remove all dust and were shade dried in open airy place with no direct sunlight. After complete exhaustion of the water contents from plant, fully dried parts were crushed to powder. This powdered plant material was subjected to successive extraction using N-hexane, ethyl acetate, methanol, and distilled water as solvents. Solvents from the extracts were removed under vacuum in a rotary evaporator at 40°C and fully dried samples were tested via preliminary biological screening. Ethyl acetate (IPT-EA, IPA-EA) and methanol (IPT-M, IPA-M) extracts from tuber and aerial part, respectively, showed maximal phytoconstituents and significant antioxidant activity which was the selection criteria for further* in vitro* and* in vivo* experimentation.

### 2.2. Chemicals and Reagents

All chemicals used in experimentation were of analytical grade purchased from different venders: BPA (Merck KGaA Darmstadt Germany), LSH and FSH (Erba Fertikit, Germany), Estradiol benzoate, Progesterone, Tween 80, ascorbic acid, 2,2- diphenyl-1-picrylhydrazyl, thiobarbituric acid (TBA), bromophenol blue (dye), ferric chloride (FeCl_3_) and aluminum chloride, ABTS, potassium persulphate, trichloroacetic acid (TCA), N-methylphenazonium methosulfate, and nitro blue tetrazolium (Sigma Chemicals Co. St. Louis, USA) and disodium phosphate (DSP), sodium dihydrogen phosphate, hydrogen peroxide (H_2_O_2_), sodium hydroxide, and sodium nitrite. Potassium ferricyanide, sulphuric acid, and ferrous chloride were obtained from Merck KGaA, Darmstadt, Germany.

### 2.3. Phytochemical Analysis

#### 2.3.1. Qualitative Assessment

*(1) Assessment of Phenols and Flavonoids*. Qualitative investigation for the traces of phenols and flavonoids was carried out by following standard procedure of Trease and Evans [25]. Suspending 1 mg of aliquote (sample) in 2 ml off distilled water (DW) enriched with 10% Iron III chloride (FeCl_3_) developed blue or green color confirming presence of phenols. Similarly mixture of extract sample (1 mg) with 2 N sodium hydroxide (1 ml) producing yellow color as end results assures presence of flavonoids.

*(2) Assessment of Coumarins and Saponins*. Presence of coumarins was assured by mixing 1 mg/ml plant extract with 10% NaOH (1 ml) and development of yellow color as end result, while formation of soapy layer with vigorous shaking of mixture containing 1 mg sample and 1 ml DW confirms saponins [26].

*(3) Assessment of Terpenoids and Triterpenoids*. Mixing of 0.5 mg plant sample with 2 ml each of chloroform concentrated sulphuric acid gives red brown colored layer in the middle of two layers when terpenoids are present. Similarly development of bluish-green color on mixing 1.5 ml of plant sample with 1 ml of Liebermann-Burchard Reagent (conc.H_2_SO_4_ + acetic anhydride) confirms presence of triterpenoids [25].

*(4) Qualitative Assessment of Tannins and Quinones*. Development of dark blue or greenish black color by mixing aliquot (1 mg) and 2 ml of 5% FeCl_3_ is confirmation for the presence of tannins in the sample, while red color as end product by mixing sample (1 mg/ml) to conc: sulphuric acid confirms quinones [25].

*(5) Qualitative Assessment of Anthocyanin and Betacyanin*. Presence of anthocyanin and betacyanin in plant samples was qualitatively assessed using established protocol of Trease and Evans [25]. By boiling mixture of 1 mg/ml solution of plant sample in 2 ml NaOH (1 N) for ten minutes, if bluish-green color is developed, it confirms anthocyanins are present. And if yellow color develops, it assures that sample is rich in betacyanin.

#### 2.3.2. Quantitative Analysis

*(1) Quantification of Total Phenolic and Flavonoid Contents*. The total phenolic contents (TPC) were estimated using Folin–Ciocalteu reagent as illustrated by Fatima et al. [27] and expressed as *μ*g gallic acid equivalent per mg dry extract (*μ*g GAE/mg extract). Similarly quantifying flavonoids method followed by Majid et al. [28] was adopted with minor modifications. Quercetin was used as standard to quantify equivalent flavonoid content in the samples and expressed as *μ*g QE/mg extract.

*(2) High Performance Liquid Chromatography (HPLC) Analysis*. Diode-Array Detection aided HPLC (HPLC-DAD) fingerprinting for the polyphenol's detection and quantification was performed adopting established protocol earlier followed by Zahra et al. [29]. External standard method was used to quantify detected polyphenols by integration of the peaks.

### 2.4. Antioxidant Profiling

Each plant sample with varying concentrations (0-500 *μ*g/ml) was used to evaluate its* in vitro* antioxidant potential. Standard protocol [28] was adopted to gauge antioxidant capacity of IPT-EA, IPA-EA, IPT-M, and IPA-M extracts. Multimode assays like DPPH scavenging, nitric oxide inhibition, hydroxyl radical inhibition, and iron chelation capacity assay were performed. The % inhibition of free radicals was calculated as

(1) $$\begin{array}{c} \%\;\;scavenging\;\;activity=1-\frac{\left(OD\;\;of\;\;sample\right)}{\left(OD\;\;of\;\;control\right)}\times 100 \end{array}$$

Reducing power and total antioxidant capacity was determined by the methodology illustrated by Ahmed et al. [30]. The results were expressed as *μ*g quercetin equivalent per mg of extract (*μ*g QE/mg).

### 2.5. Animal Ethical Statement

Guidelines of ethical committee (Quaid-i-Azam University, Islamabad, Pakistan) were strictly followed to conduct* in vivo* studies. Letters for animal care (Letter No. QAU-PHM-017/2016) and experimentation (Letter No. QAU-PHM-023/2016) were duly approved by ethical committee (dated 24/10/2016). This study plan strictly followed the guidelines that the use of animals for the experimentation should significantly provide new knowledge or lead to animal's wellbeing with least distress, pain, and discomfort to them, providing appropriate sedation, analgesia and anesthesia. Rats were euthanized by cervical dislocation under chloroform anesthesia.

### 2.6. Toxicity Assessment

#### 2.6.1. DNA Protection Assay

Genoprotective capacity of plant extracts was evaluated by conducting assay of DNA protection by previously reported method [31]. According to protocol 0.5 *μ*g/3 *μ*l of Plasmid DNA (pBR322 Fermentas) was treated with 5 *μ*l of sample (100 *μ*g/ml). Fenton reaction was induced by mixing 30% (v/v) hydrogen peroxide (4 *μ*l) with 3 *μ*l ferrous sulphate (2 mM). Control DNA (untreated), treated DNA with 2 mM FeSO_4_, 30% H_2_O_2_ + DNA, and DNA treated with 2 mM FeSO_4_ and 30% H_2_O_2_ were run at the same time as control. The incubation period for reaction mixture was 60 min at 37°C. 3 *μ*l of loading dye (Bromophenol blue) was added right after incubation to each reaction mixture. Samples were run on 1% (w/v) agarose gel loaded with ethidium bromide and TBE buffer and visualized with Syngene InGenius3 gel documentation system. Photo excitation of samples was avoided by performing experimentation in dark.

#### 2.6.2. Acute Toxicity Valuation in Rats

To investigate single booster dose effect of extracts, assay of acute toxicity was conducted. Male Sprague Dawley rats were arranged in sets representing controls and test groups. Each group contained five rats. Five doses (100, 300, 500, 1000, and 2000 mg/kg) were fed orally to the respective test groups as single booster dose and examined for fortnight for development of any toxic effects. Study was targeted to notice appearance of any visual toxic effects, mortality, behavioral changes like aggression, lethargy, loss of appetite, and balance, defecation, urination, and sleep. Increase in bodily secretions like salivation, nasal secretions, lacrimation, or any type of physical injuries was also observed during test period. Control group received single oral dose of normal saline (10 ml/kg). Guidelines 425 of Organization for Economic Cooperation and Development (OECD) were strictly followed during the study.

### 2.7. Experimental Design

Standardized conditions (12 h light/dark cycle, 25±1°C temperature) were provided to the experimental animals (*Rattus norvegicus*) weighing approximately 150-200 g/each. All test animals were properly fed and supplied with plentiful fresh water. For experimentation, 16 groups of animals (7 rats/each) were set accordingly. Specified doses (300 mg/kg and 150 mg/kg) of IPT-EA, IPA-EA, IPT-M, and IPA-M extracts, vehicle (10% DMSO), and positive (sildenafil 10 mg/kg) were administered using gastric cannula, while BPA (50 mg/kg) was injected intraperitoneally. The study plan has two major portions, i.e., behavioral study to evaluate aphrodisiac ability of* I. batatas* and its gonado protection assessment through BPA intoxication.

*Group I*. Control group remained untreated.

*Group II*. Vehicle group received 10% DMSO in water, administered orally at 10 ml/kg body weight (BW) on alternate days.

*Group III*. Positive group received sildenafil 10 mg/kg BW dissolved in 10% DMSO, given orally on alternate days.

*Group IV*. BPA group received 50 mg/kg BW dissolved in 10% DMSO, injected intraperitoneal on alternate days.

*Group V*. IPT-EA group received 300 mg/kg in 10% DMSO given orally on alternate days.

*Group VI*. IPT-M group received 300 mg/kg in 10% DMSO given orally on alternate days.

*Group VII*. IPA-EA group received 300 mg/kg in 10% DMSO given orally on alternate days.

*Group VIII*. IPA-M group received 300 mg/kg in 10% DMSO given orally on alternate days.

*Group IX*. BPA+IPT-EA group received 300 mg/kg (50 mg/kg BPA injected intraperitoneal + 300 mg/kg IPT-EA administered orally on alternate days).

*Group X*. BPA+IPT-EA group received 150 mg/kg (50 mg/kg BPA injected intraperitoneal + 150 mg/kg IPT-EA administered orally on alternate days).

*Group XI*. BPA+IPT-M group received 300 mg/kg (50 mg/kg BPA injected intraperitoneal + 300 mg/kg IPT-M administered orally on alternate days).

*Group XII*. BPA+IPT-M group received 150 mg/kg (50 mg/kg BPA injected intraperitoneal + 150 mg/kg IPT-M administered orally on alternate days)

*Group XIII*. BPA+IPA-EA group received 300 mg/kg (50 mg/kg BPA injected intraperitoneal + 300 mg/kg IPA-EA administered orally on alternate days).

*Group XIV*. BPA+IPA-EA group received 150 mg/kg (50 mg/kg BPA injected intraperitoneal + 150 mg/kg IPA-EA administered orally on alternate days).

*Group XIII*. BPA+IPA-M group received 300 mg/kg (50 mg/kg BPA injected intraperitoneal + 300 mg/kg IPA-M administered orally on alternate days).

*Group XIII*. BPA+IPA-M group received 150 mg/kg (50 mg/kg BPA injected intraperitoneal + 150 mg/kg IPA-M administered orally on alternate days).

Protocol illustrated by Ola-Mudathir et al. [32] was followed and the study was conducted for 21 days.

#### 2.7.1. Behavioral Assessment

For the evaluation of sexual behavior, 12 groups of the experimental design were used. Group I served as control, Group II as vehicle, Group III as positive control, and Group IV as negative control (BPA group) and Group V-XII were given 300 mg/kg and 150 mg/kg of IPT-EA, IPA-EA, IPT-M, and IPA-M extracts accordingly, on alternate days for 21 days. Eightyfour female rats (n=7) were brought to heat cycle (estrous) by the sequential dosing of estradiol benzoate (10 *μ*g/100 g BW) and progesterone (0.5 mg/100 g BW) via subcutaneous injections, 48 h and 4 h, respectively, before pairing [33]. After one hour of dosing, rats were paired in separate room and their sexual behavior was monitored. Before bringing female to the male cages, adaptation period of 20 min was given to the male rats to get familiar with environment.

#### 2.7.2. Assessment of Sexual Parameters

The following male sexual behavior parameters were calculated on days 1, 7, 14, and 21 after monitoring for 1 h:

*(i) Mount Latency (ML)*. The time interval between the introduction of the female and the first mount by the male.

*(ii) Intromission Latency (IL)*. The time interval between the introduction of the female and the intromission by the male.

*(iii) Mount Frequency (MF)*. The number of mounts from the time of introduction of the female until ejaculation.

*(iv) Intromission Frequency (IF)*. The number of intromissions from the time of introduction of the female until ejaculation.

*(v) Ejaculatory Latency (EL)*. The time interval between the first intromission and ejaculation. This is characterized by longer, deeper pelvic thrusting and slow dismount followed by a period of inactivity.

*(vi) Postejaculation Interval (PEI)*. The time interval between an ejaculation and the next intromission [34].

#### 2.7.3. Collection of Organ and Blood Sample

By the end of the treatment rats were kept unfed for next 24 hours. Afterwards, rats of each group were euthanized in chloroform rich environment. Blood was collected to separate serum for analysis and epididymis was cut out for weight and sperm analysis. Excised testes from various groups were homogenized in 10X buffer (100 mM potassium phosphate buffer mixed with 1 mM EDTA having pH 7.4). Homogenates were then centrifuged at 12000×g at 4°C for half an hour. Supernatant material was collected carefully and stored for use in biochemical assays. A portion of testes was excised and preserved using 10% formalin solution to use for histological studies.

#### 2.7.4. Gonadal Size, Weight, and Relative Organ Weight Determination

At the final day of the study rats of each group were weighed and sacrificed under anesthesia. The testes were excised, cleared of adhering connective tissues. Then their size was measured using Vernier caliper and weighed. Relative organ weight of the testes was found by the following formula.

(2) $$\begin{array}{c} ROW=\frac{AOW}{BW}X100 \end{array}$$

ROW stands for relative organ weight, AOW stands for absolute organ weight (g), and BW stands for body weight on final day (g).

#### 2.7.5. Epididymal Sperm Assessment

Epididymal sperm count and sperm progressive motility were evaluated by the method followed by Ola-Mudathir et al. [32]. Accordingly, epididymal spermatozoa were obtained by mincing the epididymis with anatomical scissors and 0.9 ml of physiological saline was mixed with 0.1 ml of epididymal fluid and incubated at 32°C for 2 min. An aliquot of this solution was placed in Neubauer hemocytometer and motile sperms were counted by using microscope at 40X magnification. Nonmotile sperm numbers were first determined, followed by counting of total sperm. Sperm motility was expressed as a percent of motile sperm of the total sperm counted.

#### 2.7.6. Hormone Quantification

Quantification serum testosterone concentrations were estimated using Astra Biotech kit (Immunotech Company). Sensitivity of the kit is 0.2 nmol/L–50 nmol/L. LH, FSH and estradiol were purchased from Erba Fertikit, Germany. These hormones were measured via immunoenzymatic method using ELISA reader. The experiment was performed according to the instruction [35].

#### 2.7.7. Quantification of Endogenous Antioxidant Enzymes

The supernatant from the testicular homogenates was subjected to several biochemical assays. Activities of catalase (CAT) were evaluated by monitoring the rate of H_2_O_2_ hydrolysis at 240 nm using methodology followed by Sajid et al. [36]. Superoxide dismutase (SOD) and nitric oxide (NO) inhibition activity in homogenate were gauged by standard procedure [37]. Similarly to estimate peroxidases (POD) levels in homogenates, method described by Phull et al. [38] was used with minor modifications, while reduced glutathione (GSH) was estimated by methodology adopted by Shah and Khan [39].

#### 2.7.8. Comet Assay

To estimate DNA variations, standard protocol of Dhawan et al. [40] was adopted.

DNA mutilation was evaluated by following the protocol of Dhawan et al. [40]. For 1st layering, about 3/4th part of sanitized slides was dipped in 1% solution of normal melting point agarose (NMPA) and was allowed to fix at room temperature. A tiny portion of testes was minced in 1 ml cold lysing solution and mixed with 85 *μ*l of low melting point agarose (LMPA) solution. This lysate solution containing tissue with LMPA is gently applied to precoated slides and covered cover slip. These slides were then allowed to cool for 10-12 minutes on ice bags. Then after complete drying, cover slips were removed and 2nd coating of LMPA was applied. Coated slides were again placed on ice packs to solidify media. After 3rd and final coating with LMPA, slides were exposed to lysing solution for a period of 10 min and frozen for 120 min. After 2 hours of freezing, electrophoreses were performed and slides were stained with 1% ethidium bromide dye and visualized under fluorescent microscope. CASP 1.2.3.b software for image analysis was used to calculate DNA damage. At average 50–100 cells were observed in each sample for comet parameters (head length, comet length, tail moment, tail length, and amount of DNA in head) of gonadal cell's nuclei.

#### 2.7.9. Histopathological Study of Tissues

At last day of the study plan (21st day), rats were sacrificed. Then testes of the rats were excised and a portion of it was fixed by exposure of 12 hr to 10% buffered formaldehyde (pH 7.4). This tissue sample was washed repeatedly with ethanol to remove traces of water. 5 *μ*m thick tissue slice was cut using rotary microtome and stained with Eosin and Haematoxylin (H &E staining). These stained slides were then observed under Nikon Microscope (Eclipse 80i, Japan) for histomorphological variations following the parameter evaluated by Sajid et al. [41].

### 2.8. Statistical Analysis

Data obtained in this study was presented as Mean±SD. One-way analysis of variance was performed to determine the variability among groups by Statistix 8.1. CASP 1.2.3.b software was used for comet analysis and Graph Pad Prim 5 to construct different graphs. Tukey's multiple comparison and Kruskal-Wallis tests were used to calculate significant differences among groups at P < 0.05.

## 3. Results

### 3.1. Phytochemical Evaluation

#### 3.1.1. Qualitative Analysis

The results of phytochemical analysis of IPT-EA, IPA-EA, IPT-M and IPA-M extracts are listed in Table 1A (additional data). Qualitative analysis of IPT-EA, IPA-EA, IPT-M, and IPA-M extracts ensured the presence of phenols, flavonoids, tannins, anthocyanin, saponins, and coumarins in all extracts except terpenoids which were absent in IPA-M and triterpenoids in IPT-M. Betacyanin was absent in tuber part while presence of quinones was not observed in any of the extracts. IPA-EA showed to contain the maximum classes of phytoconstituents and IPT-M with minimal phytochemical classes.

#### 3.1.2. Quantitative Analysis

**Figure 1:**
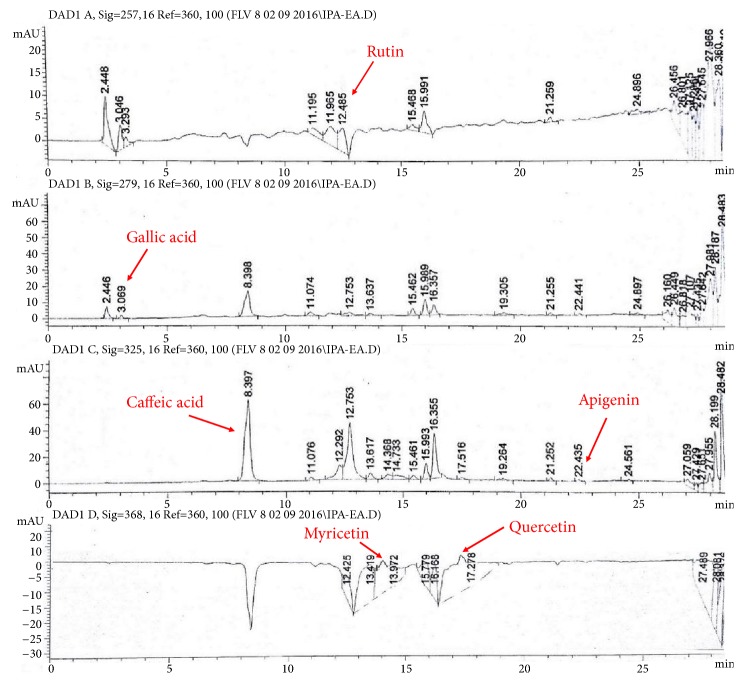
HPLC-DAD profile of* I. batatas* aerial part ethyl acetate extract (IPA-EA) at different wavelengths. Signal 1: 257*λ*, Signal 2: 279*λ*, Signal 3: 325*λ*, Signal 4; 368*λ*. Conditions: mobile Phase A-ACN:MEOH:H2O:AA/5:10:85:1, mobile phase B-ACN:MEOH:AA/40:60:1, injection volume 20 *μ*l, flow rate 1 ml/min, and agilent RP-C8.

Considering the standard regression lines for gallic acid (y = 0.0083x+0.0182; R^2^ = 0.9766) and quercetin (y = 0.0088x+0.0151; R^2^ = 0.9922), TPC and TFC quantification was performed (Table 1). Different extracts of* I. batatas *showed TPC in order of IPA-EA>IPT-EA>IPA-M>IPT-M. Similarly maximum flavonoids were quantified in IPA-EA followed by IPT-EA, IPA-M, and IPT-M. RP-HPLC based detection and quantification of polyphenols in IPT-EA, IPA-EA, IPT-M, and IPA-M extracts were performed by comparing their retention time and UV spectra with reference compounds (Figure 1). Traces of rutin, gallic acid, catechin, caffeic acid, apigenin, myricetin, and quercetin were identified and quantified in different extracts of* I. batatas*. Maximal quantity of rutin was detected in IPT-EA (7.3±0.12 *μ*g/mg dry extract) and caffeic acid in IPA-EA (4.05±0.22 *μ*g/mg dry extract) whereas apigenin was minimum in IPT-M. All the results are tabulated as Table 2.

### 3.2. *In Vitro* Antioxidant Activities

**Figure 2:**
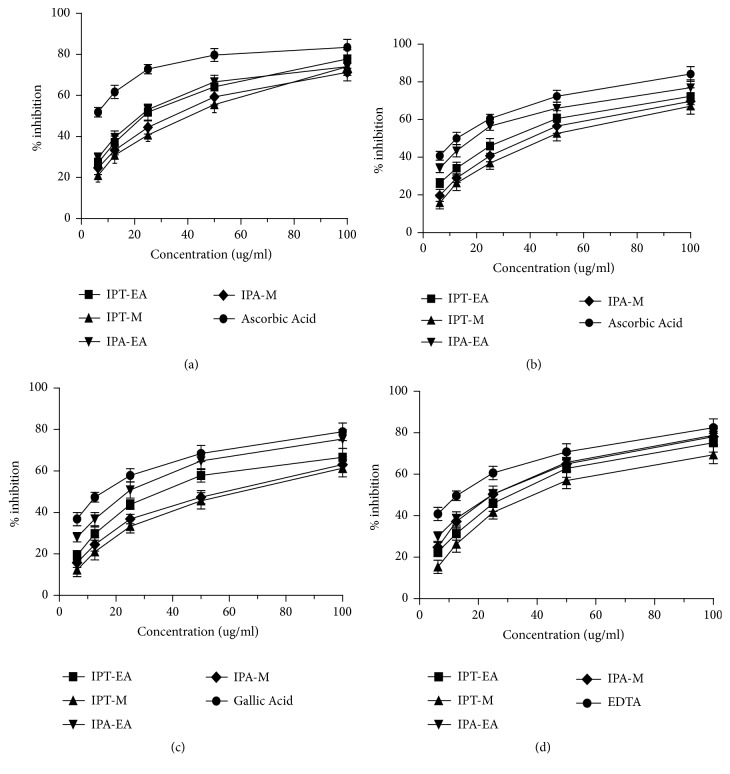
*In vitro* antioxidant activities assessment: (a) DPPH radical scavenging activity, (b) nitric oxide scavenging activity, (c) hydroxyl radical scavenging activity, and (d) iron chelating % inhibition. Each value represents Mean±SD (n=3).

Dose dependent antioxidant activity in various* in vitro* antioxidant assays was exhibited by* I. batatas* extracts (Figure 2).* I. batatas* showed antioxidant capacity in different assays in the order of nitric oxide scavenging > iron chelating ability > DPPH free radical scavenging > hydroxyl radical (OH^−^) scavenging. Total antioxidant capacity by plant extracts was greater than its total reducing power as shown in Table 1.

### 3.3. DNA Protection Capacity

**Figure 3:**
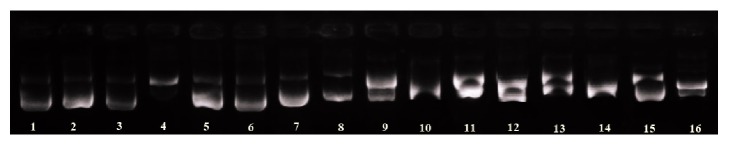
Genotoxicity evaluation of* I. batatas* extracts on pBR322 DNA using gel electrophoresis. (1), Control, (2), FeSO_4_, (3), H_2_O_2_, (4), FeSO_4_+ H_2_O_2_, (5), IPT-EA, (6), IPT-M, (7), IPA-EA, (8), IPA-M, (9), (FeSO_4_+ H_2_O_2_)+IPT-EA (300 mg/kg), (10), (FeSO_4_+ H_2_O_2_)+IPT-EA (150 mg/kg) IPT-M, (11), (FeSO_4_+ H_2_O_2_)+IPT-M (300 mg/kg), (12), (FeSO_4_+ H_2_O_2_)+IPT-M (150 mg/kg), (13), (FeSO_4_+ H_2_O_2_)+IPA-EA (300 mg/kg), (14), (FeSO_4_+ H_2_O_2_)+IPA-EA (150 mg/kg), (15), (FeSO_4_+ H_2_O_2_)+IPA-M (300 mg/kg), and (16), (FeSO_4_+ H_2_O_2_)+IPA-M (150 mg/kg).

In plasmid DNA (pBR322) genetic variation assessment using gel electrophoresis, the super coiled plasmid circular DNA moves lower in the gel showing deeper bands and the band moving slower corresponds to the open circular form (Figure 3). Herein FeSO_4_ and H_2_O_2_ treatment individually showed two bands but the native form of DNA was more concentrated, indicating a minor damaged form of DNA. But, FeSO_4_+H_2_O_2_ treated DNA showed only one band, expressing a damaged form, while IPT-EA, IPA-EA, IPT-M, and IPA-M showed dose dependent protection against Fenton induced DNA degradation. Double bands with concentrated DNA in native form show that plant extracts produced few nicks but most of the DNA was protected and super coiled which depicts genoprotective aptitude of the plant.

### 3.4. Acute Toxicity Investigations

As a result of acute toxicity assessment, plant extracts did not show any significant findings regarding variation in behavioral pattern, toxicity, or death incident during this time period. Doses ranging from 100 to 2000 mg/kg of rats were found to be safe as no deaths of the experimental groups occurred with no visible signs of toxicity observed. So these extracts were declared as nontoxic and safe to check their additional pharmacological potential within described range.

### 3.5. Aphrodisiac Activity

Aphrodisiac activity was conducted to check the potential of IPT-EA, IPA-EA, IPT-M, and IPA-M extracts. Obvious chasing of male rats towards the females in all the groups was observed, with precopulatory behaviors such as pursuing and/or anogenital sniffing leading to mounting, intromission, and ejaculation. No significant indication of tiredness was observed which was an obvious manifestation that the extract did not produce sedative effect throughout the observatory period.

#### 3.5.1. Effects on Mount Latency and Intromission Latency

**Figure 4:**
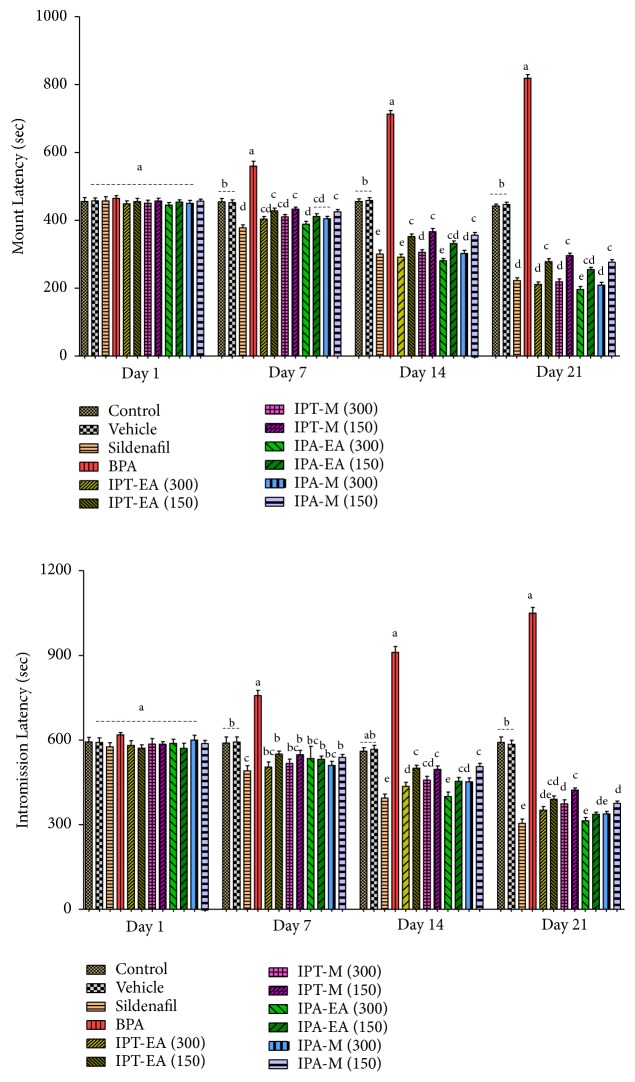
Effect of* I. batatas *on latency of mount and intromission latency. IPA-EA.* I. batatas *aerial-ethyl acetate extracts, IPA-M* I. batatas *aerial-methanol extract, IPT-EA.* I. batatas* tuber-ethyl acetate extract, and IPT-M.* I. batatas *tuber-methanol extract. Mean±SD (n=7), means with different superscript letters (^a-e^) on bars specify significant difference at P < 0.05.

The mean mount latency was gradually increasing in the BPA group of rats throughout the period of experiment, while the mount latency of the rats administered with sildenafil, IPT-EA, IPT-M, IPA-EA, and IPA-M extracts decreased from days 1 to 21. The rats in normal and vehicle showed no significant change in the latency of mounting. The IPA-EA (300 mg/kg) extract significantly decreased the latency of mount especially when compared with the control, vehicle, sildenafil, and BPA groups (Figure 4). There was a general decrease in the mean IL with the passage of time that was statistically significant (P < 0.05) when compared with control, vehicle, sildenafil, and BPA groups (Figure 4).

#### 3.5.2. Effects on Mount Frequency and Intromission Frequency

**Figure 5:**
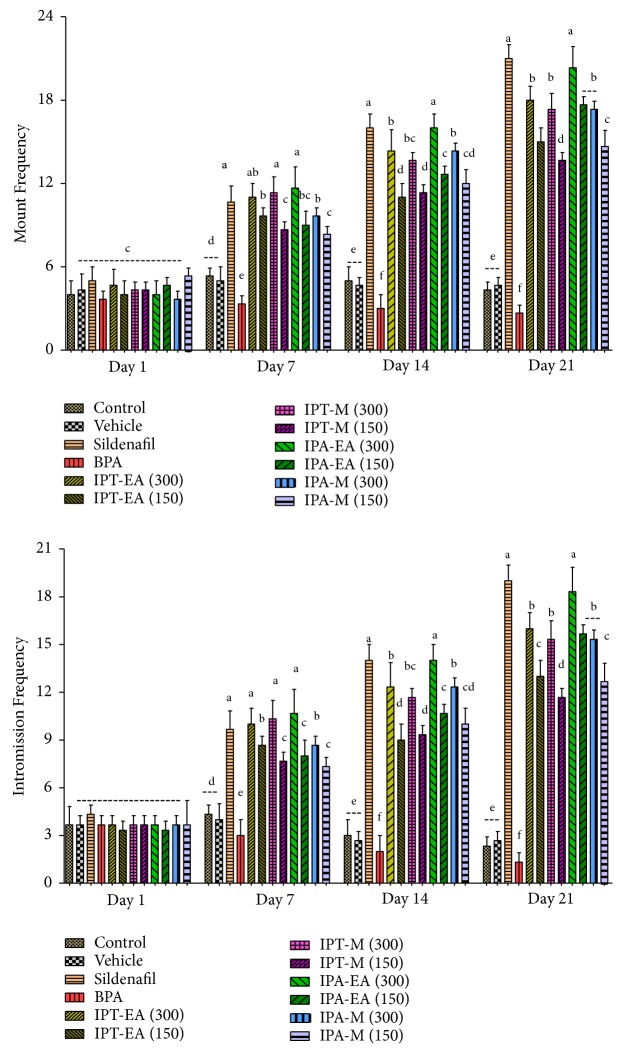
Effect of* I. batatas *on frequency of mount and intromission frequency. IPA-EA.* I. batatas *aerial-ethyl acetate extracts, IPA-M* I. batatas *aerial-methanol extract, IPT-EA.* I. batatas* tuber-ethyl acetate extract, and IPT-M.* I. batatas *tuber-methanol extract. Mean±SD (n=7), means with different superscript letters (^a-f^) on bars specify significant difference at P < 0.05.

The highest mount frequency (MF) was recorded in the rats administered with sildenafil (10 mg/kg body weight), followed by IPA-EA and IPT-EA (300 mg/kg), and the least in BPA group with gradual decrease till the end of the study. Control and vehicle showed no significant change throughout the specified time tenure (Figure 5). In this study, IPA-EA showed significant increase (P < 0.05) in mount frequency from the 1st day to day 21 in comparison to sildenafil. In a similar way IPA-EA (300 mg/kg) showed the highest frequency of intromission comparable to sildenafil while intromission frequencies decrease was statistically significant (P < 0.05) in BPA group when compared with control and vehicle (Figure 5).

#### 3.5.3. Effects on Ejaculatory Latency and Postejaculation Interval

**Figure 6:**
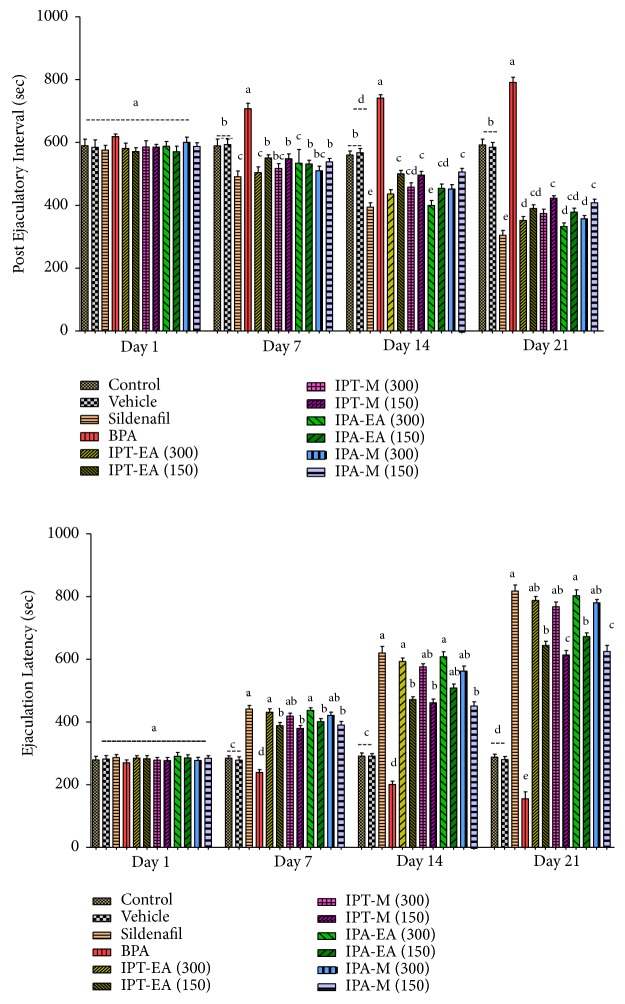
Effect of* I. batatas *on latency of ejaculation and postejaculation interval. IPA-EA.* I. batatas *aerial-ethyl acetate extracts, IPA-M* I. batatas *aerial-methanol extract, IPT-EA.* I. batatas* tuber-ethyl acetate extract, and IPT-M.* I. batatas *tuber-methanol extract. Mean±SD (n=7), means with different superscript letters (^a-e^) on bars specify significant difference at P < 0.05.

The EL of the rats administered with BPA (50 mg/kg) was significantly lower (P < 0.05) from the control and vehicle. The EL of rats administered with IPT-EA, IPA-EA, IPT-M, and IPA-M extracts increased since 1st day of administration till end of the study. The highest EL was shown by sildenafil followed by IPA-EA, IPT-EA, IPA-M, and IPT-M extracts (Figure 6), while IPT-EA stood highest among* I. batatas *extracts to decrease PEI on the final day comparable to sildenafil followed by IPA-EA. However, those rats administered with BPA (50 mg/kg) showed significant increase in PEI from days 1 to 21 as compared to control and vehicle (Figure 6).

### 3.6. Effect on Size, Weight, and Relative Organ Weight of Testes

At the end of experiment, testes of dissected rats were excised and measured. As illustrated in Table 3, the size and weight of the test group IPA-EA (300 mg/kg) have significantly (P < 0.05) increased (36.4±1.5 mm and 2.96±0.16 g, respectively) and epididymis (0.501±0.018 g) with ROW (1.37) as compared to control (23.3±2.5 mm and 2.25±0.13 g) and epididymis and ROW (0.459±0.005 g and 1.05±0.12, respectively). The BPA-induced gonadal toxicity is obvious as BPA group lost the maximum size and weight of testes (17.7±2.0 mm and 1.68±0.29 g, respectively) and epididymis (0.334±0.017 g) with ROW (0.96±0.06). Maximum protection has been examined in dose dependent manner where IPA-EA (300 mg/kg) showed significant increase in testicular size and weight and epididymal weight proving aphrodisiac and gonadoprotective effect in comparison to sildenafil as positive control (Table 3).

### 3.7. Effect on Sperm Quality

**Figure 7:**
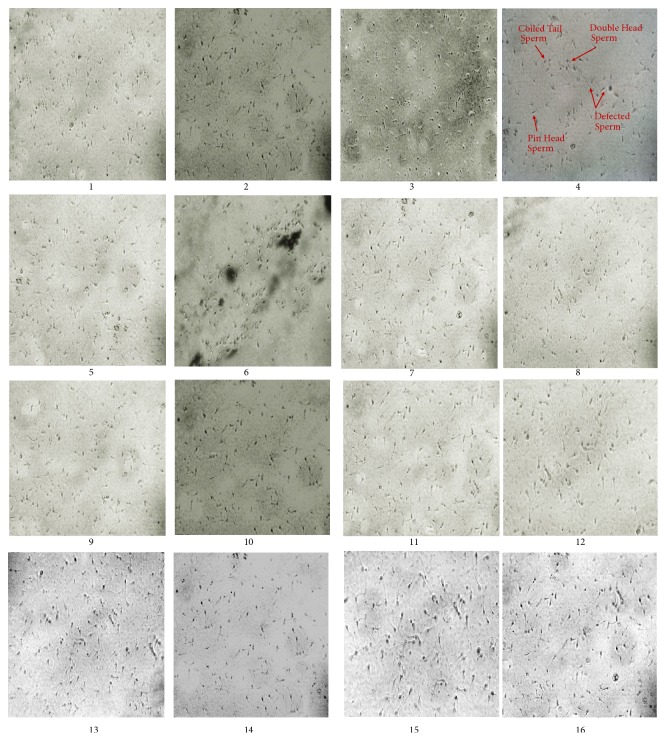
Assessment of effect of* I. batatas *on male rat's gonads. Microphotograph of rat sperms at 40X showing their morphology and count (n=7). (1), Control, (2), Vehicle, (3), sildenafil, (4), BPA, (5), IPT-EA, (6), IPT-M, (7), IPA-EA, (8), IPA-M, (9), BPA+IPT-EA (300 mg/kg), (10), BPA+IPT-EA (150 mg/kg), (11), BPA+IPT-M (300 mg/kg), (12), BPA+IPT-M (150 mg/kg), (13), BPA+IPA-EA (300 mg/kg), (14), BPA+IPA-EA (150 mg/kg), (15), BPA+IPA-M (300 mg/kg), and (16), BPA+IPA-M (150 mg/kg).

Epididymal sperm concentration, sperm motility, sperm viability, and dead/demorphed cell count is given in Table 4. Significant (P < 0.05) decrease in sperm count, % motility, and viability has been recorded in the group treated with BPA (50 mg/kg) with the highest percentage (33.9±0.06%) of dead/demorphed cells. Here IPA-EA, IPT-EA, IPT-M, and IPA-M extracts only (300 mg/kg) showed significant improvement in the sperm characteristics almost comparable to sildenafil. Test group (BPA+IPA-EA 300 mg/kg) provided maximum protection against BPA-induced gonadotoxicity and increased the concentration as well as quality of the sperms (Figure 7).

### 3.8. Effect on Hormonal Levels

Significant (P < 0.05) increase in concentrations of the circulating FSH, LH, and estradiol has been recorded in serum of rats treated with IPA-EA, IPT-EA, IPT-M, and IPA-M extracts only (300 mg/kg), comparable to sildenafil. Maximum FSH and LH (17.18±0.91 and 5.79±0.13 mIU/ml, respectively) with testosterone (6.81±0.10 ng/ml) were recorded in serum of IPA-EA (300 mg/kg) group and lowest estradiol (17.99±0.04 ng/ml) in IPT-EA (300 mg/kg) extract group in comparison to controls and vehicle. The group treated with BPA showed the lowest serum concentrations of FSH, LH (5.71±0.31 and 1.41±0.14 mIU/ml respectively), testosterone (1.47±0.06 ng/ml), and raised levels of estradiol (26.19±0.16 ng/ml) clearly indicating gonadotoxicity. Test group (BPA+IPA-EA 300 mg/kg) on the other hand showed maximum reforms in hormonal levels. Dose dependent restoration of hormones in BPA-intoxicated rats has been observed which is given in Table 5.

### 3.9. Effect on* In Vivo* Antioxidant Enzymes

Effects of vehicle, sildenafil, BPA, and* I. batatas* extracts (IPT-EA, IPA-EA, IPT-M, and IPA-M) on levels of SOD, CAT, POD, GSH, and nitric oxide in testicular homogenates are tabulated in comparison to control (Table 6). In comparison to sildenafil, IPT-EA, IPA-EA, IPT-M, and IPA-M (300 mg/kg) extracts only restored the biological concentrations of* in vivo *antioxidant enzymes and nitric oxide levels. Restoration of antioxidant enzymes and suppression of NO production was achieved in pattern: IPA-EA>IPT-EA>IPA-M>IPT-M. Maximum depletion to antioxidant enzymes level and highest NO concentrations has been recorded in BPA group which indicate ranks of toxicity to the gonads. Test groups BPA+IPA-EA (300 mg/kg) and BPA+IPT-EA (300 mg/kg) showed comparable restoration of endogenous enzymes and NO levels.

### 3.10. Effect on DNA Integrity

**Figure 8:**
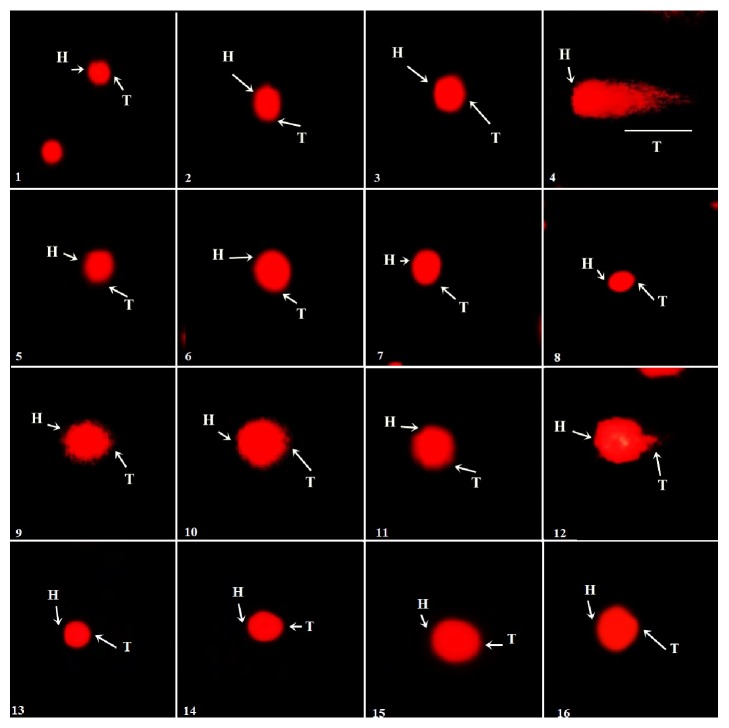
Fluorescence photomicrograph of the protective effects of* I. batatas* extracts on DNA of testes, where H is head of comet and T is tail of comet. (1), Control, (2), Vehicle, (3), sildenafil, (4), BPA, (5), IPT-EA, (6), IPT-M, (7), IPA-EA, (8), IPA-M, (9), BPA+IPT-EA (300 mg/kg), (10), BPA+IPT-EA (150 mg/kg), (11), BPA+IPT-M (300 mg/kg), (12), BPA+IPT-M (150 mg/kg), (13), BPA+IPA-EA (300 mg/kg), (14), BPA+IPA-EA (150 mg/kg), (15), BPA+IPA-M (300 mg/kg), and (16), BPA+IPA-M (150 mg/kg).

DNA protection capacities of IPT-EA, IPA-EA, IPT-M, and IPA-M extracts were tested in gametocytes as displayed in Figure 8. Comet parameters such as comet length, head length, tail length, % DNA in head, % DNA in tail, and tail moment being in complete agreement with control significantly (P < 0.05) show genoprotective nature of* I. batatas. *All the results are given in Table 7.

### 3.11. Effect on Histology of Testes

**Figure 9:**
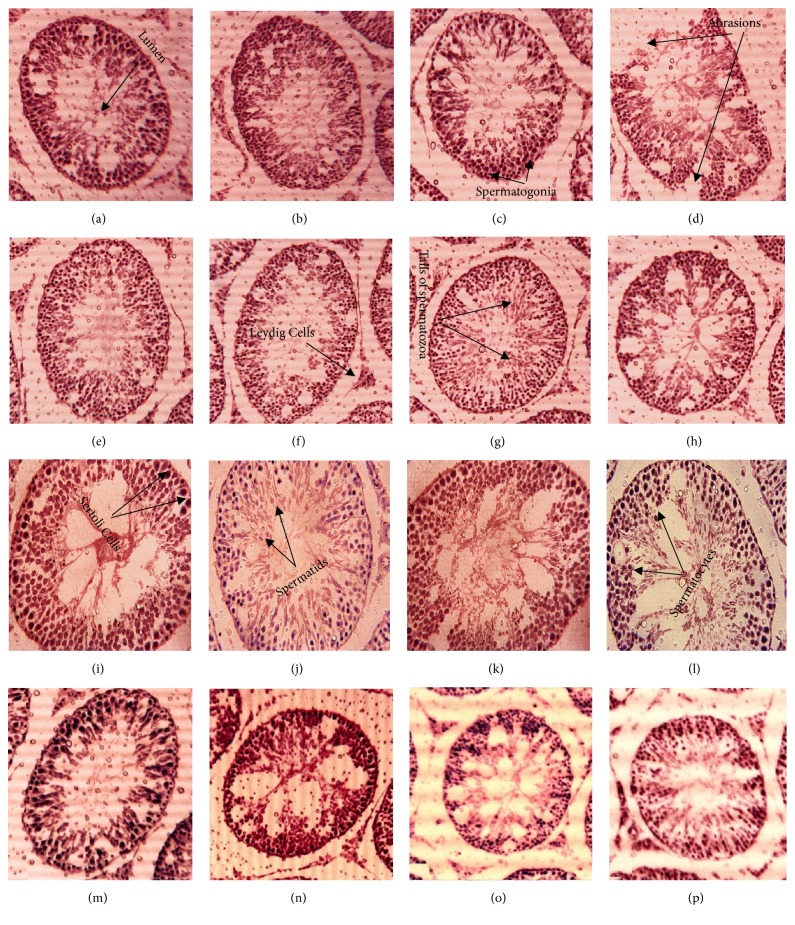
40X hematoxylin-eosin stain. Histological observations for the protective potential of* I. batatas* extracts on testes in rat. (a), Control, (b), Vehicle, (c), sildenafil, (d), BPA, (e), IPT-EA, (f), IPT-M, (g), IPA-EA, (h), IPA-M, (i), BPA+IPT-EA (300 mg/kg), (j), BPA+IPT-EA (150 mg/kg), (k), BPA+IPT-M (300 mg/kg), (l), BPA+IPT-M (150 mg/kg), (m), BPA+IPA-EA (300 mg/kg), (n), BPA+IPA-EA (150 mg/kg), (o), BPA+IPA-M (300 mg/kg), and (p), BPA+IPA-M (150 mg/kg).

Histological investigations render* I. batatas* as gonadoprotective and validating the results of comet assay. Slides significantly proved high dose (300 mg/kg) and low dose (150 mg/kg) of IPT-EA, IPA-EA, IPT-M, and IPA-M extracts which are proactive in BPA-induced toxicity (Figure 9). The control group displayed normal morphology of testes with spermatocytes, spermatids, spermatogonium, cells of Sertoli and Leydig cells, normal architecture of seminiferous tubules, normal developmental stages, and concentration of sperms in the seminiferous tubules. Test groups showed marked protection in terms of morphology of the seminiferous tubules and high density of germ cells while BPA caused significant damage and abrasions to seminiferous tubules with low cellular density.

## 4. Discussion

Aphrodisiacs are substances that stimulate or increase sexual desire and sexual performance. Hunt for effective aphrodisiacs to attain long lasting sexual powers has been a constant pursuit since time immemorial [42]. Plants like* Piper guineense, Fadogia agrestis, Aframomum melegueta, Bulbine natalensis, Lepidium meyenii, Apium graveolens (Celery), Terminalia catappa, Turnera diffusa, Allium tuberosum, Cnestis ferruginea, *and* Curculigo orchioides *have been used since centuries to cope with sex and fertility problems [1]. Various types of phytoconstituents sanction virtues and potential of the medicinal plants to play protective role against many types of diseases provide strength to the body. Previous data shows that terpenoids [43], polyphenols [44, 45], vitamins [46], *β*-carotene [47], caffeic acid [48], anthocyanin [49], and zinc [50] have gonadoprotective and spermatogenic properties. So presence of such divine medicinal groups of phytochemicals in* I. batatas *is landmark of its aphrodisiac and gonadoprotective ability.

The present study describes aphrodisiac and sexual stimulant activities of* I. batatas *extracts and their gonadoprotective capacity against bisphenol A induced toxicity in Sprague Dawley male rats.

Qualitative phytochemical screening of IPT-EA, IPT-M, IPA-EA, and IPA-M extracts confirms the presence of phenols, flavonoids, tannins, anthocyanin, saponins, coumarins, terpenoids, and triterpenoids. Presence of such divine phytoconstituents sanction* I. batatas *supreme antioxidant potential as these groups of compounds make plant capable as hydrogen donor, singlet oxygen quencher, and potent reducing agent [51]. Quantitative analysis shows that IPT-EA, IPT-M, IPA-EA, and IPA-M extracts yielded TPC (286.68±4.90, 229.45±5.01, 304.32±7.20, and 251.89±5.70 *μ*g GAE/mg DE, respectively) and TFC (188.89±2.40, 146.27±2.80, 214.77±4.09, and 170.81±2.50 *μ*g QE/mg DE, respectively) which justifies the medicinal implication of the food plant. HPLC-DAD aided significant quantification of rutin, caffeic acid, catechin, gallic acid, apigenin, myricetin, and quercetin in extracts is additional indication for therapeutic knack of this food plant. Rutin, quercetin, and gallic acid are acknowledged and well alleged secondary metabolite of plants with admirable role in gonadoprotection and improving quality of sperm [52, 53]. Similarly complementary antioxidant potential shown by plant extracts is due to these biologically proficient polyphenols. These polyphenols added considerable antioxidative capacities to* I. batatas *extracts to scavenge blends of oxidants.

To check the genoprotective abilities of* I. batatas *extracts DNA protection studies on pBR322 plasmid DNA were conducted. Hydroxyl radicals were generated by Fenton reaction to cause base alteration, deoxyribose fragmentation, and strand breakage of DNA. Polyphenols, tannins, and terpenoids in plant extracts scavenge the free radicals, decrease ROS stress, donate hydrogen to OH radical to convert it into water molecule, and irreversibly bind to the active sites of Fe^+2^ to make it inert, hence preventing Fenton reaction to complete. In the present study, the pBR322 DNA treated with Fenton reagent resulted in fragmented DNA. Marked DNA protection was observed for IPT-EA, IPT-M, IPA-EA, and IPA-M extracts, as much of the DNA content was intact and circular and moved downward in the gel. Our study is in agreement with the findings of Manikandan et al. [54] who reported protective effects of* Azadirachta indica* leaf fractions against H2O2-induced oxidative damage to pBR322 DNA and red blood cells.

Mount latency (ML) and intromission latency (IL) are indicators of sexual motivation with inverse relation. Lower ML and IL time (sec) means high sex drive and vice versa. Mount frequency (MF) and intromission frequency (IF) are valuable keys of strength, libido, and power. While the number of mounts (MF) reveals sexual motivation, increase in the number of intromission (IF) shows the penile orientation, efficiency of erection, and the ease by which ejaculatory reflexes are activated. Similarly perpetuation of the ejaculatory latency (EL) by itself suggests an aphrodisiac action and postejaculation interval (PEI) is viewed as an index of libido, potency, and the rate of retrieval from exhaustion after first series of coupling [55]. In the present study, increase in mount frequency (MF) and intromission frequency (IF) following the administration of IPT-EA, IPT-M, IPA-EA, and IPA-M extracts was observed which indicates enhanced sexual vigor/libido and sustained penile erections in the rats with improved sexual potency. The substantial increase in EL complies that the extracts extended the duration of coitus, which is an indicator of increase in sexual motivation. Also decreased PEI was observed which indicates libido enhancement, improvement of erectile function, and the ability to perform enhanced copulation. As* I. batatas* is rich in polyphenols, vitamins, proteins, and iron, the blood circulation within reproductive organs resulting in persistent erections was enhanced. Similarly, polyphenols, terpenoids, tannins, anthocyanin, and zinc significantly enhance spermatogenic capacity. Enhanced copulatory performance in rats administered with extracts is confirmation that* I. batatas* is aphrodisiac in nature. On the other hand, rats administered with BPA showed decrease in the frequency of mounting and intromission and increased hesitation time of the male rats towards the receptive females clearly indicating gonadotoxicity in those rats which ultimately caused depressed sex drive. Chauhan et al. [56] reported that ethanol extract of seeds of* Bryonia laciniosa* Linn administered orally to groups of male albino rats at the dose levels of 50, 100, and 150 mg kg^−1^ body weight per day for 28 days significantly enhanced sexual vigor and copulatory performance.

Spermatic production can be quantified by measuring the testicular weight and size and weight of epididymis, as testes are mainly composed of seminiferous tubules and its high volume indicates increased spermatogenesis. FSH and LH are called gonadotropins as they stimulate the gonads (testes) in males. Within testes, FSH binds with estrogen receptor *α* in the Sertoli cells and LH binds to estrogen receptor *β* in Leydig cells and increases production of testosterone and stimulates spermatogenesis. Testosterone maintains normal sexual desire, nocturnal penile tumescence, and nonerotic penile erections in most men [57, 58]. Oral treatment of rats with polyphenol rich* I. batatas *extracts and sildenafil significantly increased size, weight, and relative weight of testes and epididymis in comparison to the control and vehicle group which clearly indicates the high spermatic production due to increase in length of seminiferous tubules and proliferation of germ cells. Similarly raised levels of serum FSH, LH, and testosterone and lowered estradiol level indicate enhanced spermatogenesis. BPA has good fat solubility and hence can easily cross blood brain barrier and effect brain-gonads-pituitary gland axis function, therefore, disturbing development of reproductive organs, testosterone excretion, and spermatogenesis [59]. Rats given BPA showed expressive decrease in size, weight, and relative weight of testes and epididymis which clearly suggest high level of testicular toxicity. Similarly in BPA-intoxicated testes proliferation of Sertoli and Leydig cells causes destruction of gonadotropin-releasing hormone (GnRH) and inhibin which are necessary for the negative feedback of FSH, LH, and ultimately testosterone influencing production of gonadotropins and testosterones. Test groups with oral treatment of* I. batatas *along with intraperitoneal administration of BPA showed prominent recovery of testicular toxicity because polyphenols and terpenoids are good at reversing ROS induced gonadotoxicity. Also flavonoids, terpenoids, and trace elements especially zinc vitalize central nervous system ultimately improve sexual potency. High levels of gonadotropins and testosterones are validating the behavioral parameters being investigated. Comparable investigations have been conducted by Shah and Khan [39] on* Jurinea dolomiaea* extract in rat model.

BPA-induced testicular injuries are associated with depleted amount of endogenous antioxidant enzymes and raised nitrite production. Endogenous antioxidant enzymes (CAT, SOD, POD, and GSH) provide a first line of defense inside the body against ROS. As ROS concentration rises inside the body, expression of these enzymes is increased to cope with the stressed situation. No significant change in CAT, SOD, and POD has been calculated in groups treated with* I. batatas *(300 and 150 mg/kg) and sildenafil (10 mg/kg) in comparison to the control and vehicle. Because polyphenol rich* I. batatas *provided a strong defense against blends of free radicals and mitigated the oxidative stress, raised nitrite levels in BPA-intoxicated rats are the indication of injuries to the vascular endothelium or the activation of neutrophils in damaged testicular tissue which caused synthesis of NO. Similarly levels of GSH are found raised in test groups as glutathione is present in high amounts on the surface of the sperm and increased sperm motility [60, 61]. Hence increased spermatogenesis might be the reason of elevated expression of GSH. Increased levels of GSH and low NO concentration are validating the high percentage of sperm motility and viability in test groups while vice versa for BPA group. Previously extract of* Pistacia chinensis *have been reported to have similar aptitude and raised the GSH level in testes [7].

Male reproductive system comprises a composite arrangement of Sertoli cells, Leydig cells, and epididymal and germ cells in which each one is substantial member of spermatogenesis and critical for male fertility. Continuous exposure of genetic material to endocrine disrupting chemical (EDC) like BPA causes raised oxidative stress, hypomethylation, mutations, testicular disruption, and strand breakage of sperm DNA resulting immature or demorphed spermatozoa [62]. To check BPA-induced toxicity in testicular morphology and genome and genoprotective aptitude of* I. batatas*, testicular histology and comet assay have been conducted. Cross section of seminiferous tubules has shown marked toxicological effects of test groups treated with BPA showing destructed Leydig cells, desorbed seminiferous tubules, and profuse round spermatids while* I. batatas *showed significant protection (Figure 9). Similarly large amount of genetic content in comet tail (Figure 8) is indication of DNA fragmentation induced by BPA while comet tail was much smaller proving DNA protective aptitude of the extracts. As generation of ROS is considered to be involved in the oxidative DNA damage, polyphenol rich* I. batatas* extracts provide solid defense line by decreasing the ROS level and DNA fragmentation and increasing the cell viability. The nontoxic nature of plant sanctioned its safety and suitability as alternative aphrodisiac source with satisfactory outcomes. Kazmi et al. [63] have conducted similar type of genoprotective study on* Q. dilatata.*

## 5. Conclusion

Complete scheme of behavioral and* in vivo *studies in rat model declared that* I. batatas *(IPT-EA, IPT-M, IPA-EA, and IPA-M) extracts are capable of coping with infertility problems and BPA-induced gonadotoxicity. Significant stimulation in sexual behavior, elevated spermatic production, raised viability, optimal gonadal hormones production, maintained endogenous enzymes, genoprotection, and reformed testicular histology endorsed plant as a better aphrodisiac alternative with significant phytochemical and antioxidant profile. Still mechanism based studies on molecular levels are needed for optimal verification.

## Figures and Tables

**Table 1:** Total phenolic contents, total flavonoid contents, total antioxidant capacity, and total reducing power of *I. batatas *extracts.

Samples	TPC(*μ*g GAE/mg DE)	TFC(*μ*g QE/mg DE)	TAC(*μ*g QE/mg DE)	TRP(*μ*g QE/mg DE)
	y = 0.0083x + 0.0182	y = 0.0088x + 0.0151	y = 0.0133x+0.036	y = 0.0144x+0.0349
IPT-EA	286.68±4.90^b^	188.89±2.40^b^	442.48±4.85^b^	332.48±4.06^b^
IPT-M	229.45±5.01^d^	146.27±2.80^d^	361.65±3.35^d^	256.09±4.56^d^
IPA-EA	304.32±7.20^a^	214.77±4.09^a^	453.71±4.26^a^	349.67±5.28^a^
IPA-M	251.89±5.70^c^	170.81±2.50^c^	388.15±5.95^c^	294.71±4.42^c^

TPC: total phenolic contents, TFC: total flavonoid contents, TAC: total antioxidant capacity, TRP: total reducing power, GAE: gallic acid equivalent, QE: quercetin equivalent, and DE: dry extract. Data values shown represent Mean±SD (n=3). Means with different superscript (a-d) letters in the column are significantly (P < 0.05) different from one another.

**Table 2:** HPLC-DAD analysis of *I. batatas *extracts.

Flavonoids/Phenolics	Signal wavelength	Quantity (*μ*g/mg dry extract)			
	(*λ*)	IPA-EA	IPA-M	IPT-EA	IPT-M
Rutin	257	1.57±0.17^c^	1.62±0.18^c^	7.3±0.12^a^	0.75±0.08^c^
Gallic acid	279	0.27±0.08	0.28±0.05	0.24±0.080	nd
Catechin	279	nd	1.19±0.15^c^	0.74±0.04^c^	nd
Caffeic acid	325	4.05±0.22^b^	1.92±0.17^c^	1.60±0.15^c^	0.52±0.07^c^
Apigenin	325	0.26±0.09	0.23±0.06	0.21±0.040	0.18±0.04
Myricetin	368	1.32±0.11^c^	1.50±0.11^c^	2.7±0.14^b^	nd
Quercetin	368	0.65±0.06	0.72±0.09	0.45±0.05	nd
Kaempferol	368	nd	nd	nd	nd

IPA-EA: *I. batatas *aerial-ethyl acetate extracts, IPA-M: *I. batatas *aerial-methanol extract, IPT-EA: *I. batatas* tuber-ethyl acetate extract, and IPT-M: *I. batatas *tuber-methanol extract. Each value is represented as Mean±SD (n=3). ^a, b, c^  represents the significance of flavonoid/phenolic quantified and ^nd^  stands for not detected.

**Table 3:** Assessment of size, weight, and relative organ weight of testes.

Sample	Size (mm)	Weight of whole epididymis (g)	Weight of both testes (g)	Final Body Weight (g)	ROW
Control	23.3±2.5^b^	0.459±0.005^de^	2.25±0.13^b^	213±8.0^c^	1.05±0.12^b^
Vehicle (10% DMSO)	22.8±1.2^b^	0.456±0.012^de^	2.23±0.18^b^	208±11^bc^	1.07±0.07^b^
Sildenafil (10 mg/kg)	36.0±3.5^g^	0.479±0.009^e^	2.88±0.21^j^	217±5.0^cd^	1.32±0.12^e^
BPA (50 mg/kg)	17.7±2.0^a^	0.334±0.017^a^	1.68±0.29^a^	174±13.0^a^	0.96±0.06^a^
IPT- EA (300 mg/kg)	34.7±1.8^f^	0.478±0.008^e^	2.78±0.11^i^	221±9.0^d^	1.25±0.04^d^
IPT-M (300 mg/kg)	31.7±1.1^e^	0.470±0.012^e^	2.66±0.09^h^	217±8.0^cd^	1.22±0.06^cd^
IPA-EA (300 mg/kg)	36.4±1.5^g^	0.501±0.018^f^	2.96±0.16^k^	216±6.0^cd^	1.37±0.08^f^
IPA-M (300 mg/kg)	35.2±0.7^f^	0.476±0.006^e^	2.74±0.13^hi^	213±12^c^	1.28±0.35^de^
BPA+IPT-EA (300 mg/kg)	28.4±1.4^d^	0.421±0.003^c^	2.53±0.14^f^	205±12^b^	1.23±0.11^cd^
BPA+IPT-EA (150 mg/kg)	25.1±0.9^c^	0.402±0.011^bc^	2.37±0.04^d^	202±18^b^	1.17±0.15^c^
BPA+IPT-M (300 mg/kg)	26.1±1.7^cd^	0.408±0.005^bc^	2.42±0.12^de^	201±8.0^b^	1.20±0.18^cd^
BPA+IPT-M (150 mg/kg)	24.9±1.1^c^	0.389±0.012^b^	2.31±0.11^c^	208±12^bc^	1.11±0.10^bc^
BPA+IPA-EA (300 mg/kg)	30.9±1.1^e^	0.449±0.007^d^	2.71±0.16^h^	210±12^bc^	1.29±0.16^de^
BPA+IPA-EA (150 mg/kg)	28.1±1.2^d^	0.416±0.009^c^	2.51±0.14^f^	205±15^b^	1.22±0.13^cd^
BPA+IPA-M (300 mg/kg)	28.8±1.3^d^	0.419±0.005^c^	2.60±0.21^g^	203±7.0^b^	1.28±0.08^de^
BPA+IPA-M (150 mg/kg)	26.2±1.5^cd^	0.396±0.014^bc^	2.45±0.17^e^	200±20^b^	1.22±0.11^cd^

ROW, relative organ weight, BPA, bisphenol A, IPT, *Ipomoea batatas *tuber, and IPA, *Ipomoea batatas* aerial part. Data values represent Mean±SD (n=7). Means with different superscript (^a-k^) letters in the column are significantly (P < 0.05) different from one another.

**Table 4:** Effects of *I. batatas *on sperm characteristics.

Sample	% Motility	% Viability	Dead/demorphed cells (%)	Sperm count
				(×10^6^/ml)
Control	76.2±2.5^c^	82.5±2.3^b^	5.02±0.04^ab^	83.13±1.78^ab^
Vehicle (10% DMSO)	75.7±1.2^c^	81.8±2.8^b^	6.14±0.07^b^	82.65±2.45^b^
Sildenafil (10 mg/kg)	80.4±3.5^ab^	86.3±2.2^a^	6.44±0.12^b^	84.92±2.82^ab^
BPA (50 mg/kg)	37.7±4.4^g^	47.6±2.9^f^	33.9±0.06^g^	48.23±4.09^g^
IPT- EA (300 mg/kg)	80.1±3.8^ab^	84.1±1.8^ab^	4.39±0.04^ab^	85.13±1.78^ab^
IPT-M (300 mg/kg)	79.4±1.4^b^	82.3±2.4^b^	5.31±0.11^ab^	83.29±3.11^ab^
IPA-EA (300 mg/kg)	82.8±1.1^a^	87.5±1.9^a^	3.99±0.26^a^	87.31±1.26^a^
IPA-M (300 mg/kg)	76.5±2.3^c^	79.7±2.2^bc^	5.2±0.18^ab^	84.22±3.14^ab^
BPA+IPT-EA (300 mg/kg)	71.1±1.5^d^	74.2±1.6^c^	11.8±0.08^d^	74.43±4.85^d^
BPA+IPT-EA (150 mg/kg)	66.5±3.3^e^	69.5±1.9^d^	14.2±0.12^de^	68.12±2.78^e^
BPA+IPT-M (300 mg/kg)	67.9±1.1^e^	71.3±1.1^cd^	15.4±0.11^e^	72.71±2.91^de^
BPA+IPT-M (150 mg/kg)	61.2±3.5^f^	66.3±2.2^e^	19.8±0.18^f^	66.43±4.15^ef^
BPA+IPA-EA (300 mg/kg)	74.2±1.7^cd^	78.6±1.3^bc^	8.37±0.05^c^	77.28±3.53^c^
BPA+IPA-EA (150 mg/kg)	67.1±2.5^e^	72.2±1.7^cd^	12.3±0.10^d^	72.43±2.40^de^
BPA+IPA-M (300 mg/kg)	69.4±2.4^de^	74.9±2.2^c^	13.3±0.09^de^	72.98±3.47^de^
BPA+IPA-M (150 mg/kg)	62.7±1.9^f^	68.5±1.5^de^	17.8±0.15^ef^	65.13±2.05^f^

Mean±SD (n=7), means with different superscript letters (^a-g^) in a column specify significant difference at P < 0.05. IPA, *I. batatas* aerial and IPT, *I. batatas* tuber extract.

**Table 5:** Appraisal of reforms in hormonal levels by *I. batatas*.

Sample	Testosterone (ng/ml)	FSH (mIU/ml)	LH (mIU/ml)	Estradiol (ng/ml)
Control	4.41±0.12^c^	11.27±0.52^c^	3.14±0.23^c^	18.02±0.14^a^
Vehicle (10% DMSO)	4.33±0.09^c^	10.93±0.75^c^	3.29±0.33^c^	19.14±0.17^b^
Sildenafil (10 mg/kg)	6.89±0.14^a^	16.68±0.89^a^	5.85±0.28^a^	18.10±0.22^a^
BPA (50 mg/kg)	1.47±0.06^f^	5.71±0.31^f^	1.41±0.14^e^	26.19±0.16^f^
IPT- EA (300 mg/kg)	6.05±0.11^ab^	14.66±0.82^b^	4.74±0.32^b^	17.99±0.04^a^
IPT-M (300 mg/kg)	5.54±0.15^b^	14.14±0.54^b^	4.34±0.23^b^	18.88±0.11^ab^
IPA-EA (300 mg/kg)	6.81±0.10^a^	17.18±0.91^a^	5.79±0.13^a^	18.33±0.16^a^
IPA-M (300 mg/kg)	5.78±0.12^b^	14.05±0.43^b^	4.45±0.18^b^	19.26±0.28^b^
BPA+IPT-EA (300 mg/kg)	3.98±0.06^cd^	9.21±0.35^d^	2.71±0.12^cd^	21.88±0.18^cd^
BPA+IPT-EA (150 mg/kg)	3.69±0.12^de^	8.31±0.22^de^	2.38±0.10^d^	23.18±0.23^e^
BPA+IPT-M (300 mg/kg)	3.82±0.05^d^	8.59±0.18^de^	2.54±0.09^cd^	22.14±0.11^cd^
BPA+IPT-M (150 mg/kg)	3.56±0.10^e^	7.87±0.29^e^	2.31±0.15^d^	23.21±0.18^e^
BPA+IPA-EA (300 mg/kg)	4.22±0.09^c^	10.2±0.27^cd^	3.09±0.13^c^	20.07±0.17^bc^
BPA+IPA-EA (150 mg/kg)	3.93±0.16^cd^	9.22±0.36^d^	2.59±0.12^cd^	21.28±0.08^de^
BPA+IPA-M (300 mg/kg)	3.87±0.11^d^	9.04±0.42^d^	2.65±0.10^cd^	21.39±0.12^c^
BPA+IPA-M (150 mg/kg)	3.58±0.12^e^	8.01±0.35^e^	2.35±0.09^d^	22.78±0.31^d^

FSH, follicle stimulating hormone and LH, luteinizing hormone. All the data are represented as Mean±SD (n=7), means with different superscript letters (^a-f^) in a column specify significant difference at P < 0.05. IPA, *I. batatas* aerial and IPT, *I. batatas* tuber extract.

**Table 6:** Effect of *I. batatas *on endogenous antioxidant enzyme and NO levels.

Sample	CAT (U/mg protein)	POD (U/mg protein)	SOD (U/mg protein)	GSH (*μ*M/mg protein)	Nitrite *μ*M/mg protein)
Control	3.77±0.12^a^	9.14±0.63^b^	18.02±1.54^b^	24.81±3.14^c^	56.78±2.34^a^
Vehicle (10% DMSO)	3.83±0.09^a^	9.29±0.39^b^	19.14±1.37^a^	23.68±2.91^d^	57.23±4.58^a^
Sildenafil (10 mg/kg)	3.61±0.11^ab^	9.85±0.28^a^	18.44±1.62^ab^	26.92±4.25^a^	57.57±4.27^a^
BPA (50 mg/kg)	1.71±0.07^f^	3.41±0.14^g^	8.19±1.56^h^	12.71±2.84^j^	83.54±3.12^j^
IPT- EA (300 mg/kg)	3.66±0.12^ab^	8.94±0.32^c^	17.59±1.44^bc^	25.65±4.72^b^	58.12±2.89^ab^
IPT-M (300 mg/kg)	3.42±0.10^b^	8.47±0.23^cd^	17.21±1.11^c^	23.89±5.28^d^	59.01±3.26^b^
IPA-EA (300 mg/kg)	3.73±0.06^a^	9.21±0.43^b^	18.23±0.86^ab^	26.34±4.77^a^	56.34±3.51^a^
IPA-M (300 mg/kg)	3.67±0.13^ab^	8.85±0.58^c^	17.46±1.28^bc^	24.88±3.69^c^	58.68±2.59^ab^
BPA+IPT-EA (300 mg/kg)	3.21±0.15^c^	7.71±0.32^de^	15.88±1.18^de^	19.66±4.28^ef^	67.25±1.98^d^
BPA+IPT-EA (150 mg/kg)	2.98±0.10^cd^	5.92±0.40^f^	13.11±0.74^g^	17.17±2.18^h^	73.33±1.39^g^
BPA+IPT-M (300 mg/kg)	2.89±0.18^cd^	7.54±0.29^de^	15.14±0.91^e^	19.03±3.19^f^	69.11±3.16^e^
BPA+IPT-M (150 mg/kg)	2.56±0.11^de^	5.80±0.38^f^	12.84±1.21^g^	16.60±2.10^i^	75.01±2.59^h^
BPA+IPA-EA (300 mg/kg)	3.12±0.27^c^	7.99±0.43^d^	16.67±1.45^d^	20.23±2.14^e^	63.38±2.74^c^
BPA+IPA-EA (150 mg/kg)	2.82±0.21^d^	7.01±0.21^e^	14.54±1.58^f^	18.22±2.43^g^	68.76±2.10^de^
BPA+IPA-M (300 mg/kg)	2.64±0.32^de^	7.65±0.40^de^	15.19±1.22^e^	19.45±5.12^ef^	71.87±3.15^f^
BPA+IPA-M (150 mg/kg)	2.37±0.15^e^	6.05±0.40^f^	12.96±1.06^g^	17.05±3.45^h^	76.56±2.01^i^

All the data are represented as Mean±SD (n=7), means with different superscript letters (^a-j^) in a column specify significant difference at P < 0.05. CAT, catalase, POD, peroxidase, SOD, superoxide dismutase, and GSH, glutathione.

**Table 7:** Cytotoxicity assessment on testes by comet parameters.

Sample	Comet length (*μ*m)	Head length (*μ*m)	Tail length (*μ*m)	% DNA in head	% DNA in tail	Tail moment (*μ*m)
Control	42.6±3.1	36.7±2.5	5.9±0.3	86.1±2.8	13.9±1.8^*β*^	0.11±0.03^*β*^
Vehicle (10% DMSO)	43.3±2.8	37.2±2.4	6.1±0.4	85.9±1.3	14.1±1.9^*β*^	0.12±0.03^*β*^
Sildenafil (10 mg/kg)	40.4±4.2	35.9±1.8	4.5±0.5	88.8±2.1	11.2±1.3^*β*^	0.11±0.04^*β*^
BPA (50 mg/kg)	44.6±3.6	27.1±2.7	17.5±1.4	60.1±3.5	39.9±1.8^¥^	1.27±0.11^¥^
IPT- EA (300 mg/kg)	39.5±1.2	34.6±2.5	4.9±0.7	87.5±3.7	12.5±1.4^*β*^	0.12±0.02^*β*^
IPT-M (300 mg/kg)	42.1±3.2	35.7±1.7	7.4±1.1	84.8±2.2	15.2±1.8^*β*^	0.13±0.06^*β*^
IPA-EA (300 mg/kg)	43.7±2.6	38.2±1.3	5.5±0.8	87.5±1.7	12.5±1.2^*β*^	0.11±0.03^*β*^
IPA-M (300 mg/kg)	37.6±1.9	31.5±1.5	6.1±0.6	83.8±3.6	16.2±1.1^*β*^	0.14±0.04^*β*^
BPA+IPT-EA (300 mg/kg)	41.4±2.4	31.7±1.8	9.7±1.3	76.5±1.8	23.5±2.6^*β*¥^	0.24±0.03^*β*¥^
BPA+IPT-EA (150 mg/kg)	43.9±2.8	32.4±2.9	8.5±0.9	80.6±3.1	19.4±2.5^*β*^	0.20±0.06^*β*¥^
BPA+IPT-M (300 mg/kg)	40.1±2.7	32.5±1.4	7.6±1.0	81.1±2.4	18.9±1.3^*β*^	0.16±0.04^*β*^
BPA+IPT-M (150 mg/kg)	42.4±3.3	33.9±2.1	9.4±1.2	79.9±1.5	20.1±2.4^*β*¥^	0.21±0.05^*β*¥^
BPA+IPA-EA (300 mg/kg)	37.1±1.2	31.6±1.1	5.5±0.6	85.1±3.6	14.9±1.1^*β*^	0.14±0.04^*β*^
BPA+IPA-EA (150 mg/kg)	40.3±2.8	33.5±2.9	6.8±0.9	83.1±3.1	16.9±2.5^*β*^	0.18±0.06^*β*^
BPA+IPA-M (300 mg/kg)	42.6±3.1	33.1±2.1	9.5±1.2	77.7±1.5	22.3±2.4^*β*¥^	0.20±0.05^*β*¥^
BPA+IPA-M (150 mg/kg)	41.2±3.3	31.9±2.4	9.3±1.0	77.4±1.5	22.6±1.9^*β*¥^	0.21±0.05^*β*¥^

BPA, bisphenol A, IPA, *I. batatas* aerial, and IPT, *I. batatas* tuber extract. Values are expressed as Mean±SD (n=7). Means with symbol “^*β*^” indicate nonsignificant difference from normal control; “^¥^”from BPA treated group according to Kruskal-Wallis test at P < 0.05.

## Data Availability

The data used to support the findings of this study are available from the corresponding author upon request.

## Supplementary Materials

Supplementary Materials Graphical demonstration of aphrodisiac and gonadoprotective potential of* Ipomoea batatas* against BPA-induced testicular damage. Table 1. Phytochemical analysis of* I. batatas*.
