# Supplementary material for: Scientific Validation of Ethnomedicinal Use of Ipomoea batatas L. Lam. as Aphrodisiac and Gonadoprotective Agent against Bisphenol A Induced Testicular Toxicity in Male Sprague Dawley Rats
Source: Biomed Res Int. 2019 Apr 14;2019:8939854. doi: 10.1155/2019/8939854 (PMC6487138; doi:10.1155/2019/8939854)
Supplement: Supplementary Materials — Graphical demonstration of aphrodisiac and gonadoprotective potential of Ipomoea batatas against BPA-induced testicular damage. Table 1. Phytochemical analysis of I. batatas. [file 8939854.f1.pdf]

Additional files

Additional file 1

Table 1. Phytochemical analysis of *I. batatas*

Phytochemical	IPT-EA	IPT-M	IPA-EA	IPA-M
**Phenols**	+++	++	+++	++
**Flavonoids**	+++	++	+++	++
**Coumarins**	+	+	+	++
**Saponins**	+	++	+	+
**Terpenoids**	++	+	++	-
**Triterpenoids**	++	-	+++	+
**Tannins**	+	++	++	++
**Quinones**	-	-	-	-
**Anthocyanin**	++	++	+++	++
**Betacyanin**	-	-	++	+

(+) present, (-) absent, (++) moderate concentration, (+++) abundant concentration. IPA-EA. *I. batatas* aerial-ethyl acetate extracts, IPA-M *I. batatas* aerial-methanol extract, IPT-EA. *I. batatas* tuber-ethyl acetate extract, IPT-M. *I. batatas* tuber-methanol extract.

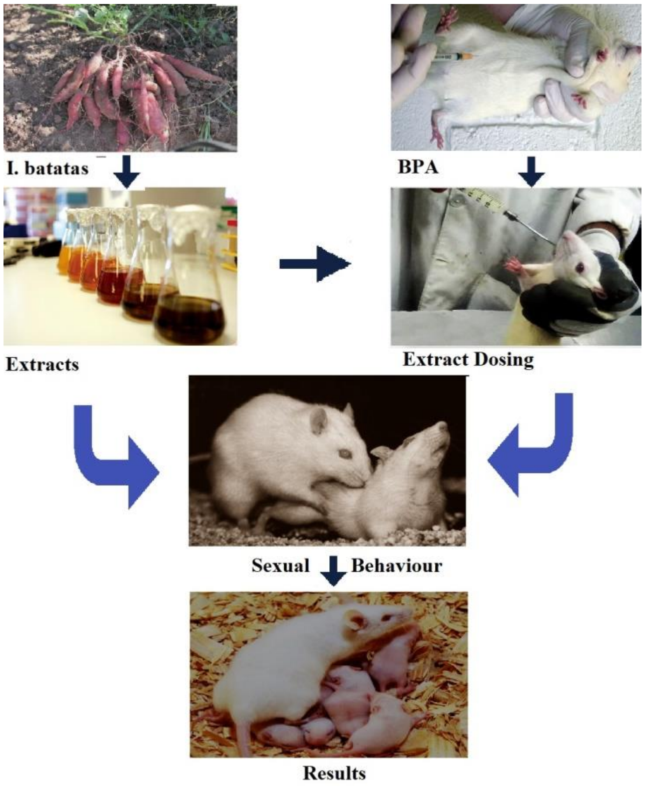

Graphical demonstration of aphrodisiac and gonadoprotective potential of Ipomoea batatas against BPA induced testicular damage
